# Stereotactic aspiration alone or Ommaya placement and aspiration followed by stereotactic radiosurgery for cystic brain metastasis: A systematic review and meta-analysis

**DOI:** 10.1016/j.bas.2025.104184

**Published:** 2025-01-11

**Authors:** David R. Peters, Alfredo Conti, Marc Levivier, Luis Schiappacasse, Mohamed Faouzi, Mioara Florentina Trandafirescu, Constantin Tuleasca

**Affiliations:** a Carolina Neurosurgery & Spine Associates, Charlotte, NC, USA; b Mayo Clinic, Rochester, MN, USA; c Lausanne University Hospital (CHUV), Neurosurgery Service and Gamma Knife Center, Lausanne, Switzerland; d University of Lausanne (UNIL), Faculty of Biology and Medicine (FBM), Switzerland; e Department of Neurosurgery, IRCCS Istituto delle Scienze Neurologiche di Bologna, Bologna, Italy; f Dipartimento di Biomorfologia e. Scienze Neuromotorie (DIBINEM), Alma Mater Studiorum Università di Bologna, Bologna, Italy; g Lausanne University Hospital (CHUV), Radiation Oncology Department, Lausanne, Switzerland; h Division of Biostatistics, Center for Primary Care and Public Health (Unisanté), University of Lausanne, Switzerland; i University of Medicine and Pharmacy “Gr. T. Popa”, Iasi, Romania; j Ecole Polytechnique Fédérale de Lausanne (EPFL, LTS-5), Lausanne, Switzerland

**Keywords:** Stereotactic radiosurgery, Brain metastases, Cystic brain metastases, Ommaya reservoir, Stereotactic aspiration, Cyst aspiration

## Abstract

**Introduction:**

Cystic brain metastases (BMs) are often more challenging to treat than solid BMs. Stereotactic cyst aspiration for volume reduction followed by stereotactic radiosurgery (SRS) is an alternative treatment modality that may benefit patients with large cystic BMs not favorable for SRS alone nor microsurgical resection.

**Research question:**

Here, we perform a systematic review and meta-analysis of stereotactic aspiration alone or reservoir (Ommaya) placement plus aspiration followed by SRS for cystic BMs.

**Material and methods:**

Using the Preferred Reporting Items for Systematic Reviews and Meta-Analyses (PRISMA) guidelines, we reviewed articles published between 1968 and December 31^−th^, 2022. We retained 10 studies reporting 280 patients.

**Results:**

Overall rate of tumor control for combined treatment of Ommaya placement plus aspiration plus SRS was 81.2% (62.5–99.9%, p < 0.001) and for stereotactic aspiration plus SRS was 64.7% (46.1–83.3%, p < 0.001). Overall rate of further intervention for combined treatment of Ommaya placement plus aspiration plus SRS was 15.8% (p = 0.08) and for stereotactic aspiration plus SRS was 14.8% (5.3–24.4%, p = 0.002). Overall complication rate for combined treatment of Ommaya placement plus aspiration plus SRS was 12.8% (2.3–23.3%, p = 0.01) and for stereotactic aspiration plus SRS was 1.5% (p = 0.12).

**Discussion and conclusion:**

Combined treatment of Ommaya placement plus cyst aspiration plus SRS in cystic BMs yields better local control as compared to stereotactic aspiration plus SRS, with similar rate of further intervention between procedures. Aspiration of the cyst plus SRS should be considered for patients with cystic metastases not able to undergo open surgery or upfront SRS.

## Highlights

• Cystic brain metastases (BMs) are often more challenging to treat than solid BMs.
• Combined treatment Ommaya placement + cyst aspiration + SRS in cystic BMs yields better local control as compared to stereotactic aspiration + SRS.
• Aspiration of the cyst + SRS should be considered for patients with cystic BM not able to undergo open surgery or upfront SRS.

## 1 Introduction

Brain metastases (BMs) constitute a major source of morbidity and mortality, with an incidence that has been estimated to range from 20 to 40% in cancer patients [ (Patchell and Regine, 2003), (Patchell, 2003)]. Without treatment, BMs are associated with poor prognosis, typically with a median overall survival of approximately 1 month [ (Lagerwaard et al., 1999), (Markesbery et al., 1978)]. Treatment options for BMs include a combination of stereotactic radiosurgery (SRS), fractionated radiotherapy, surgical resection, chemotherapy, immunotherapy, and/or whole brain radiotherapy (WBRT).

It is not unusual for BMs to develop a large, cystic, liquid component adjacent to the tumor tissue. Cystic BMs pose multiple challenges compared to solid BMs. The cyst fluid is typically acellular but can greatly increase size of the tumor [ (Pan et al., 2005)]. This can create tumors with large overall volume and significant mass effect despite having a small solid tumor component. Local control rate is inversely related to tumor volume and directly correlated to radiation dosage [ (Baschnagel et al., 2013), (Shiau et al., 1997)]. Once a tumor reaches a size >3 cm in diameter or >15 mL in volume, SRS treatment alone is usually insufficient, as prescription doses high enough for adequate local control of these large lesions typically carry an unacceptably high risk of radiation necrosis [ (Pan et al., 2005), (Kalkanis et al., 2010; Linskey et al., 2010; Lippitz et al., 2014; Kondziolka et al., 1999)]. Even if the solid tumor component is well treated, the cystic component still has the potential to grow, thereby increasing the risk of exacerbating neurological symptoms [ (Pan et al., 2005), (Hayashi et al., 2011)]. SRS alone also does not address the neurological symptoms caused by mass effect, which are frequently observed in these larger lesions.

As a result, surgical resection is typically recommended for large cystic BMs, providing both symptom relief and oncological benefit [ (Kalkanis et al., 2010)]. However, some patients are not good candidates for microsurgical resection. Deep or eloquent location, co-morbidities, advanced age, and patient preference are all factors that can make surgery less favorable. Furthermore, cystic BMs often consist of a thin ring of tumor tissue forming a capsule that surrounds a large cystic cavity. The presence of this morphology increases the difficulty of achieving a safe, gross total resection. Safely removing the tumor tissue composing the cyst walls while ensuring the preservation of the surrounding healthy brain tissue poses a greater difficulty compared to the resection of solid BMs. In addition, surgery can spread tumor cells throughout the intracranial space, increasing the risk of leptomeningeal disease [ (Press et al., 2019)]. Stereotactic cyst aspiration for volume reduction followed by SRS is a valuable alternative treatment modality that may benefit patients with large cystic BMs not favorable for SRS alone nor microsurgical resection.

Here, we performed a systematic review and meta-analysis of the current knowledge regarding the combined use of cyst aspiration plus SRS for large cystic brain metastases. We review aspiration effectiveness, local control, overall survival, complications, and current recommendations.

## 2 Methods

### 2.1 Study guidelines

The study was performed in accordance with the published Preferred Reporting Items for Systematic Reviews and Meta-Analyses (PRISMA) guidelines [ (Moher et al., 2009)].

We screened the following source: Medline, Pubmed, Embase, Scopus and Web of Science databases.

**Fig. 1:**
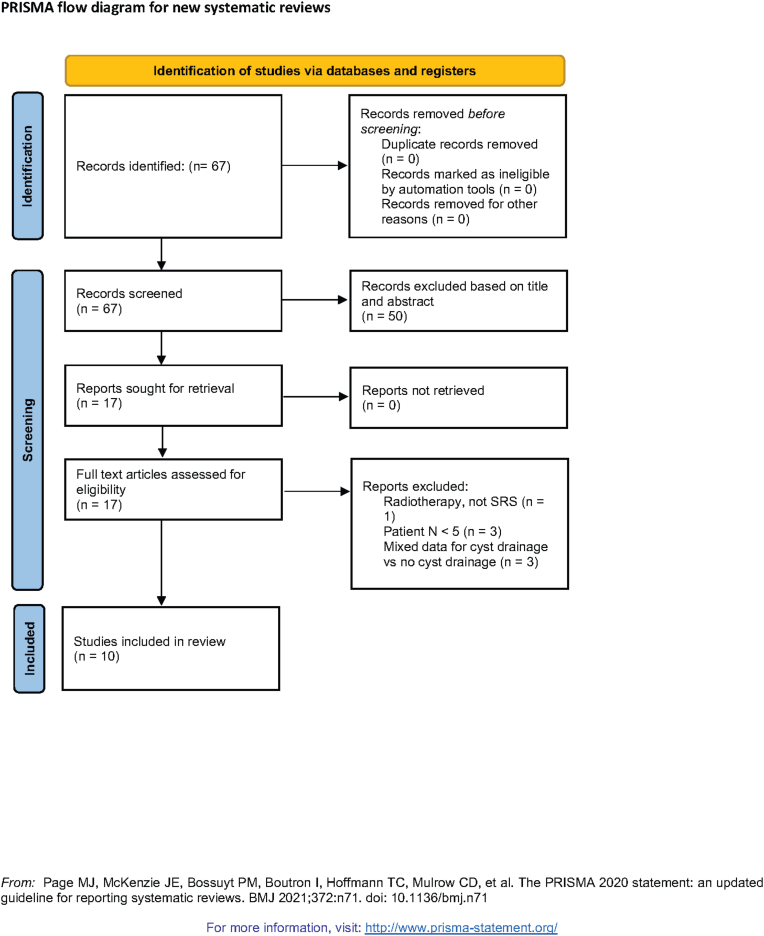
PRISMA diagram showing study selection.

We used the following MESH terms or a combination of these: “brain metastases”, “cystic”, “radiosurgery”, “surgery”, “Ommaya”, “reservoir”, “stereotactic”. Two independent reviewers (DP, CT) have screened the content of all articles and abstracts published between 1968 and December, 31^−th^ 2022. The corresponding PRISMA diagram can be found in Fig. 1.

### 2.2 Eligibility criteria

Inclusion criteria were peer-reviewed articles of intracranial cystic BM treated either with stereotactic aspiration plus SRS or reservoir (Ommaya) placement and aspiration followed by SRS.

Exclusion criteria were case reports, unpublished series, and series not published in English.

### 2.3 Article selection

Four articles (and 77 patients) met the inclusion criteria for Ommaya implantation followed by cyst aspiration plus SRS [ (Noda et al., 2022; Oshima et al., 2017; Yamanaka et al., 2006; Park et al., 2021)]. Six articles (and 203 patients) met the inclusion criteria for stereotactic aspiration plus SRS (Franzin et al., 2008, Higuchi et al., 2012, Jung et al., 2014; Park et al., 2009; Sadik et al., 2021; Wang et al., 2016).

We extracted clinical data related to patient demographics (Table 1) and outcomes (Table 2).

**Table 1:** Demographic data for large vestibular schwannomas treated with planned subtotal resection and stereotactic radiosurgery.

	Number of patients	Criteria and strategy for combined	Age mean (median; range)	Male:female	Follow-up after SRS (months)	NF2-related schwannomatosis	Surgical approach	Degree of resection	Post-microsurgical complications (other than facial and cochlear)
Iwai et al.[21] (2003)	14	Extrameatal diameter >30 mm STR + GKS	47(-;18-64)	6:8	32 (-;12-72)	2/14	R: 13/14 TP: 1/14	STR: 13/14 PR: 1/14	Cranial IV palsy: 1/14 Mortality: 0/14
Park et al.[25] (2006)	8	diameter >30 mm STR + GKS	NR	NR	68.8 (-;-)	0/8	NR	Radical subtotal removal: 8/8	NR
Fuentes et al.[49] (2008) Not reviewed	8	diameter >30 mm Planned STR + GKS	53(-;24-78)	5:3	46(-;12-73)	NR	R: 8/8	STR: 8/8	NR
Yang et al.[29] (2008) Not reviewed	61	STR + GKS	-(41; 18-67)	20:41	-(53.7;24.1-102.2)	NR	NR	STR: 61/61	NR
Van de Langenberg et al.[28] (2011)	50	Growing Koos grade III and upfront for Koos grade IV Planned STR + GKS	52(-;21-84)	28:22	- (33.8;12-84)	NR	R: 25/50 TL: 25/50	STR: 50/50	Hematoma: 2/50 Hydrocephalus: 2/50 VP shunt: 1/50 Lumbar drain: 1/50 Temporary dysfunction CN IX, X: 3/50 Transient CN VI: 1/50 Hemiparesis: 1/50
Pan et al.[24] (2012)	G1 (intracapsular decompression):18 G2 (extracapsular resection): 17	diameter >30 mm Planned STR + GKS	G1: 50 ± 3.0 G2: 49 ± 2.3	G1: 10:8 G2: 10:7	G1: 57.7 ± 3.3 G2: 52.7 ± 1.8	NR	G1: R: 18/18 G2: R: 17/17	G1: STR: 18/18 G2: STR: 17/17	G1: 1/18 CSF shunt G2: 1/17 CSF shunt
Daniel et al.[19] (2018)	47	Koos grade IV Planned STR + SRS	51.2(;22-85)	22:25	37.5 (36;0.5-96)	0/47	R: 47/47	STR: 47/47	1/47 transient hypoesthesia after surgery 1/47 CN X deficit 4/47 CSF shunt
Troude et al.[30] (2019)	169 (77 upfront SRS)	Koos grade IV STR + GKS	51(-; 16-85)	67:102	Overall: 62 (54; 11-147) 70(75) in SRS group after SRS (because “67-75” is 95% CI)	10/169	R: 108/169 TL: 61/169	GTR: 16/169 NTR: 94/169 STR: 34/169 PR: 15/169	Keratitis: 20/169; corneal ulcer: 5/169; Abducens: 6/169; IX, X: 5/169; CPA hematoma: 15/169 (10 symptomatic); CSF leak: 18/169; wound infection: 4/169; meningitis: 8/169; hydrocephalus with shunt: 4/169 Pulmonary embolism: 4/169; death: 2/169 4/169 facial numbness Lower cranial nerve: 5/77, 100% transient
Suero Molina et al.[27] (2019)	160 (148 available for clinical, 157 for radiological follow-up)	STR + GKS	-(55;14-89)	63:97	-(36;3-180)	6/160	1 surgery: 144/160 2 surgeries: 11/160 3 surgeries: 3/160 4 surgeries: 2/160	GTR: 118/146 Subcapsular resection: 28/146	1/160 CSF shunt
Ng et al.[23] (2020)	10	Koos grade IV STR + GKS	47.9 (49.7; 20.6-69.6)	5:5	7.2 (6.9; 1.6-15.5)		R: 8/10 TL: 2/10	STR: 10/10	NR
Radwan et al.[31] (2021) Not reviewed	17	Maximum diameter >40 mm, corresponding to Koos III or IV Planned STR + SRS	56(-;-)	7:15	40 (20,20-128)	NR	NR	STR: 22/22	Trigeminal neuropathy: 4/22 2/22: dysphagia, dysarthria
Iwai et al.[20] (2021) Not reviewed	47	Maximum diameter more than 25 mm	-(60;30-82)	22:25	-(74;24-180)	0/47 (personal communication)	R: 47/47	STR: 47/47	1/47 lung abscess 1/47 aseptic meningitis 1/47 pulmonary embolism 1/47 cerebellar venous infarction
Lee et al.[22] (2021)	68	Planned STR + SRS	-(42.5;14-83)	26:42	-(64;25.7-152.4)	0/69	R: 66/68 TL: 2/68	STR: 68/68	1/68 CSF leakage 1/68 cerebellar dysfunction 1/68 epidural hematoma 1/68 surgical site infection 2/68 lower cranial nerve dysfunction 3/68 CSF shunt

STR = subtotal resection; SRS = stereotactic radiosurgery; GKS= Gamma Knife surgery; NR = not reported; R = retrosigmoid approach; TL = translab approach; CSF = cerebrospinal fluid.

**Table 2:** Outcomes: local control and cranial nerves preservation rates for patients with large vestibular schwannomas treated with planned subtotal resection and SRS.

	Tumor diameter at surgery	Tumor volume at surgery	Postsurgery facial outcome	Postsurgery cochlear outcome	Interval surgery and SRS (mths)	Tumor size at SRS (mm)	Tumor volume at SRS (mL)	Marginal dose (Gy)	Tumor control; stability; decrease	Tumor increase	Post-SRS complications
Iwai et al.[21] (2003)	30-40:6/14 40-50:6/14 >50:2/14	NR	I: 10/14 II: 2/14 HB I or II: 12/14 III:2/14 HB I or II: 10/14 III or more: 4/14	1/3	2.9 (-;1-6)	18.9(-;9.8-36.1)	–	12.1 (;10-14.1)	11/14 5/14 6/14	3/14 One NF2 pt underwent salvage MS.	0/14 HB I/II: 12/14 (stable compared to surgery) Cochlear 1/3 (stable compared to surgery)
Park et al.[25] (2006)	35.4 (-;30-47.2)	26.8(-;13.5-55.1)	NR	NR	-(-;0.25-6)	NR	4.6(-;-)	12	8/8;-;-	0/8	0/8 HB I/II 7/7 Cochlear NR
Fuentes et al.[49] (2008) not reviewed	39.4 (-;35-45)	NR	7/8	NR	9(;6-12)	18(-;9-20)	1.2(-;0.3-2.2)	11.8(-;11-13)	8/8;-;-	0/8	0/8 HB I/II: 7/8 (stable compared to surgery) Cochlear NR
Yang et al.[29] (2008) not reviewed	NR	20.6 (-;4.1-44.5)	HB I or II: 58/61	5/10	5.8 (-;0.3-95.7) Starting 2000, between 4 and 6 months after surgery	NR	NR	12.5 (-;9-14)	58/61 8/61 50/61 8y: 93.5%	3/61	0/61 58/58 HB I/II 3/5 kept serviceable hearing after SRS 3/10 if considering combined
Van de Langenberg et al.[28] (2011)	35(- ;26-54)	14.9 (- ;4.1-36.1)	HB I/II: 34/50 III or more: 16/50	1/4	8.5 (- ;2-24)	NR	3.34(- ;0.22-11.8)	11(-;9.4-11.9)	45/50 16/50 29/50	5/50 Second GKS: 3/50 Second MS + SRS: 1/50	HB I/II: 47/50 (13 had transient deficit after surgery) 2/50 had transient facial nerve deficit 1/50 persistent hemifacial spasm 1/4 same as after surgery
Pan et al.[24] (2012)	NR	G1: 17.5 ± 1.1 G2: 16.4 ± 0.95	G1: HB I/II: 16/18 G2: HB I/II/III/IV: 2/4/3/8	G1: 11/11 G2: 0/11	G1: 3.6 ± 0.2 G2: 7 ± 0.4	NR	G1: 9.35 ± 1.02 G2: 1.1 ± 0.14	G1:12(12;-) G2: 12(12;-)	G1: 18/18 NR NR G2: 17/17	G1: 0/18 G2: 0/17	18/18 (2 recovered distantly from a facial palsy after surgery) G1: HB I/II: 16/2 Cochlear: 11/11 CSF shunt: 1 G2: HB I/II/III/IV: 2/4/4/7 Cochlear: 0/11 CSF shunt: 2
Daniel et al.[19] (2018)	33(31.5;20-45)	11.8 (-;1.5-34.9)	HB I: 47/47 Including 1 recovery (IV to I)	19/22	6(5;3-13.9)	NR	3.3 (-; 0.5-12.8)	12 (12;11-12)	43/47	4/47 3 Microsurgery and 1 unknown details	HB II: 1/47 3 years after SRS HB I: 46/47 19/22 hearing preservation as after surgery
Troude et al.[30] (2019)	Extrameatal 30.2(30;19-55)	16.5(14;4-87)	HB I: 122/145 HB II: 22/145 HB I/II: 144/145 HB IV: 1/145	2/19 preserved	6.8 (-;3-11)	NR	0.83(0.55;-)	12(12;-)	62/77	15 regrowth (1 FU lost, 4 Observation, 9 GKS and 1 Microsurgery)	HB I/II: 144/145 same as after surgery 2/19 cochlear preservation, same as after surgery
Molina et al.[27] (2019) Only after tumor progression	NR	-(1.4;0.06-35.8)	NR	NR	-(49;2-315)	NR	- (1.4; 0.06-35.8)	– - (13; 12-14)	136/158	22/158 after a median of 26 mths (5-56) 14/148 clinical with second SRS (6/148) or microsurgery (8/148)	At median 36 (3-180) Trigeminal: 3/148 Facial: 3/148 Cochlear: 2/149
Ng et al.[23] (2020)	NR	NR	HB I or II: 6/10 HB III: 3/10 HB VI: 1/10	NR	41.8 (23.7; 3.6-117.5)	14.9 (15.7;3.9-26.8)		12-13 Gy at the 50% isodose line	8/10 7/10 1/10	2/10 at 23 months after SRS (increased by 23% and by 37% respectively)	HB I/II: 7/10 HB III: 2/10 HB V: 1/10 Cochlear NR
Radwan et al.[31] (2021) not reviewed	Maximum diameter >40 mm	13.1(-;-)	Immediate after surgery HB I-III: 15/22 HB IV-V: 5/22 HB VI: 2/22		9.5 (7, 2-50)		2.9(-;-)	10/17 single fraction 12-14 Gy 7/17 multisession (25 Gy in 5 fractions or 21 Gy in 3 fractions)	14/17 13/17 1/17 Radiological control: 80% Oncological control: 100% Mean extent of resection was 77%	3/17 but not requiring treatment	HB I/II: 19/22 same as after surgery 7/8, after surgery 8/8 1/8 declined after SRS
Iwai et al.[20] (2021) not reviewed	-(32;25-52)	NR	HBI/II: 44/47 HB IV: 1/47 HB V: 2/47	Improved: 2/16 Preserved: 13/16	-(3;1-12)	NR	-(2.7; 0.4-10.4)	-(12;10-12)	43/47 3y: 92% 5y: 86% 10y: 86% 15y: 86%	4/47 After a median of 31 months (12-42) after SRS	HB I/II: 44/47 same as after surgery Cochlear 13/16 0/47 ARE 2/47 transient hemifacial spasm 2/47 transient trigeminal neuropathy associated with TTE
Lee et al.[22] (2021)	NR	-(15.4;3.2-40.9)	HB I/II/III/IV/V: 39/15/6/7/1	6/27 preserved	(4.2;0.7-16.2)	NR	-(2.5;0.3-27.4)	-(12.5;10-20) 1 case underwent fractionated GKS 20 Gy in 4 fractions	60/68	8/68 after a median progression time of 15.8 mths (3.2-66)	HB I/II/III/IV/V:44/13/2/7/2 (4 recovered between surgery and SRS; 2/68 aggravated facial palsy from HB II) 6/27 preserved, as after surgery Others: 3/68 hemifacial spasm

SRS = stereotactic radiosurgery; HB= House et Brackmann classification; NR = not reported; MS = microsurgery.

### 2.4 Primary and secondary outcomes

Primary outcome was tumor control. Secondary outcomes were rate of complications and need for further intervention.

### 2.5 Statistical analysis

OpenMeta (Analyst) from the Agency for Healthcare Research and Quality was used for statistical analysis. A binary random-effects model (DerSimonian-Laird method) was chosen. Weighted summary rates were identified, testing for heterogeneity was completed, and pooled estimates were attained for all the outcomes of interest.

## 3 Results

### 3.1 Pre-treatment volumes and volumes after aspiration

The pre-treatment volumes, as well as volumes after aspiration, whenever available, can be found in Table 1. The number of cyst aspirations can be also found in Table 1, and ranges from one to more than 4.

### 3.2 Radiosurgery treatment doses and mean volumes before and after SRS

Radiosurgery treatment doses and mean volumes before and after SRS can be found in Table 2. SRS treatment doses ranged from 11 to 25 Gy in single fraction; this marginal dose has been chosen by some centers as function of the maximal diameter (18 Gy for less than 10 mm, 16 Gy for 10–20 mm or 14 Gy for more than 20 mm). Other centers have used fractionated RT using a number of fractions between 3 and 10 and variable dose protocols (for more details please see Table 2).

### 3.3 Local control

**Fig. 2:**
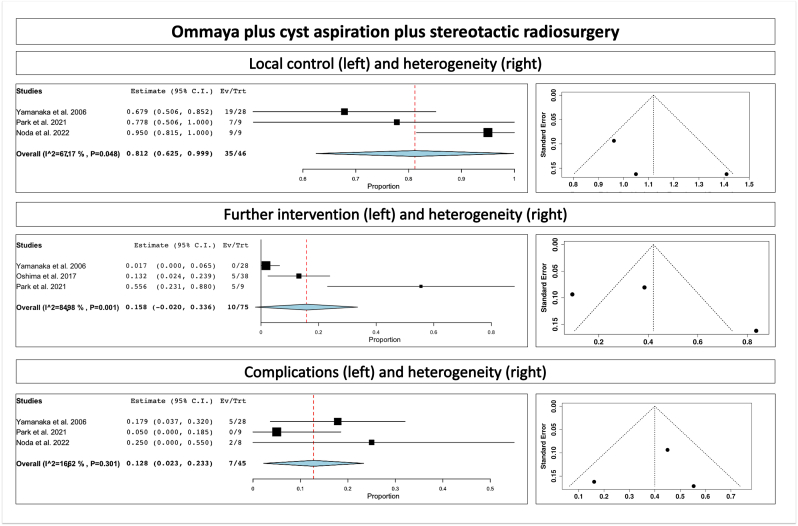
Ommaya plus cyst aspiration plus stereotactic radiosurgery, showing local control (upper part), further intervention (middle part) and complications (lower part); we assessed for heterogeneity (right side of the figure).

**Fig. 3:**
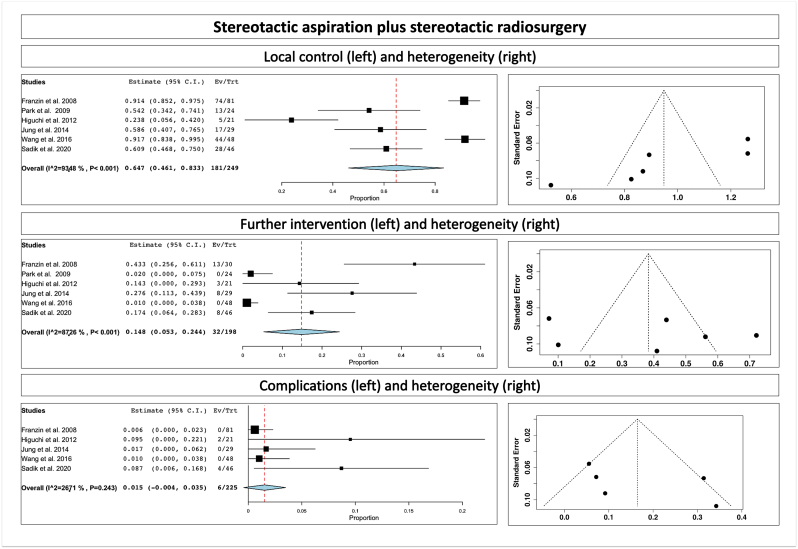
Stereotactic aspiration plus stereotactic radiosurgery, showing local control (upper part), further intervention (middle part) and complications (lower part); we assessed for heterogeneity (right side of the figure).

The overall rate of tumor control for combined treatment of Ommaya placement plus aspiration plus SRS was 81.2% (range 62.5–99.9%, p heterogeneity = 0.04, *p* < 0.001; Table 2; Fig. 2, upper part) and for stereotactic aspiration plus SRS was 64.7% (range 46.1–83.3%, p heterogeneity<0.001, *p* < 0.001; Table 2; Fig. 3, upper part). We assessed for heterogeneity (right side of the figure).

### 3.4 Further intervention

The overall rate of further intervention for combined treatment of Ommaya placement plus aspiration plus SRS was 15.8% (p heterogeneity = 0.001, *p* = 0.08; Table 2; Fig. 2, middle part) and for stereotactic aspiration plus SRS was 14.8% (range 5.3–24.4%, p heterogeneity<0.001, *p* = 0.002; Table 2; Fig. 3, middle part). We assessed for heterogeneity (right side of the figure).

### 3.5 Complications

The overall complication rate for combined treatment of Ommaya placement plus aspiration plus SRS was 12.8% (range 2.3–23.3%, p heterogeneity = 0.3, *p* = 0.01; Table 2; Fig. 2, lower part) and for stereotactic aspiration plus SRS was 1.5% (p heterogeneity = 0.24, *p* = 0.12; Table 2; Fig. 3, lower part). We assessed for heterogeneity (right side of the figure).

## 4 Discussion

Our meta-analysis suggests that the overall rate of tumor control, further intervention, and complications for combined treatment of Ommaya placement plus aspiration plus SRS and for stereotactic aspiration plus SRS were 81.2% versus 64.7%, 15.8% versus 14.8% and 12.8% versus 1.5%, respectively. In the Ommaya group, the complication rate was higher, but most were minor, unlike the stereotactic aspiration group which had fewer but more serious complications (two deaths).

Cystic changes in BMs are thought to be the result of increased permeability of pathologic vessels and mesodermal reactive processes, tumor degeneration followed by transudation of fluid from blood vessels [ (Cumings, 1950), (Stem, 1939)]. Cystic change is most common in BM from lung cancer [ (Niranjan et al., 2000), (Uchino et al., 2000)]. Cystic BMs also tend to have a less effective reaction to SRS compared to solid BMs [ (Pan et al., 2005), (Franzin et al., 2008)]. Typically, a large cystic BM is a strong indication for surgical resection since an operative intervention is the fastest way to relieve neurological symptoms caused by mass effect [ (Kalkanis et al., 2010), (Franzin et al., 2008)]. However, not all patients are good surgical candidates, and, if the mass effect can be treated without tumor resection, SRS could be used to treat the tumor tissue while stereotactic cyst aspiration is used to treat the mass effect. Combining these procedures is possible for large cystic masses, unlike large solid masses, which require surgical resection to relieve the mass effect. Aspiration of a cyst can provide immediate improvement of symptoms in 70–80% of symptomatic cystic tumors, but as much as 30% may have a recurrence of the cystic fluid and mass effect, requiring additional placement of a catheter and reservoir system[ (Niranjan et al., 2000)].

Stereotactic radiosurgery is especially important for patients with advanced systemic cancer who often must undergo further treatment systemically during their disease [ (Pan et al., 2005), (Linskey et al., 2010)]. It has now been well acknowledged that tumor volumes are inversely correlated with local control rates and overall survival [ (Pan et al., 2005), (Higuchi et al., 2012)]. According to the RTOG, for single fraction radiosurgery is prescribed 24 Gy, 18 Gy, and 15 Gy for tumors≤20 mm, 21–30 mm, 31–40 mm in maximal diameter, respectively [ (Shaw et al., 2000)]. Moreover, high prescription doses on larger target volumes have been linked with greater risk of radiation necrosis [ (Park et al., 2021)]. Tumor volumes inversely correlate with local control and survival, especially as they exceed diameter of 3 cm [ (Petrovich et al., 2002), (Varlotto et al., 2003)]. The cause for this is likely inadequate prescribed dose, as local control significant drops for tumors treated with prescribed dose of less than 20 Gy, but higher doses increase the risk of radiation necrosis [ (Han et al., 2012; Majhail et al., 2001; Shehata et al., 2004)]. Often, cystic tumors have a small solid component, but the large cystic fluid component puts the size of the tumor over the reasonable size limit for SRS. Aspiration eliminates the problem of large tumor size and acellular fluid volume for SRS. It can also alleviate mass effect and improve symptoms rapidly while limiting dissemination of tumor cells and cystic fluid. Cyst aspiration can be performed by either stereotactic aspiration alone or with placement of an Ommaya reservoir where the catheter is placed inside the cyst, allowing for serial aspiration over time as needed. Once aspiration is performed to shrink the cyst, the smaller tumor can then be treated effectively with SRS. This allows access to the advantages provided by SRS: minimal invasiveness, ability to treat multiple lesions at the same time, access to deep, surgically inaccessible lesions, and substantial reduction of hospitalization and costs, without sacrificing rates of local control [ (Mehta et al., 1997), (Rutigliano et al., 1995)]. This technique may prove particularly advantageous for cancer patients at advanced stages of the primary disease who require ongoing systemic treatment. It enables them to expedite their treatment progress without the need to wait for recovery from microsurgical resection.

Large cystic BM usually occur in the subcortical region near the cortex [ (Yamanaka et al., 2006)]. For this location, it is relatively safe and not technically challenging to perform an aspiration under CT/MRI guidance, which can be performed in a stereotactic manner or using frameless systems [ (Yamanaka et al., 2006)]. The period between aspiration and SRS should be as short as possible to better capitalize on the reduced tumor volume before cystic fluid can reaccumulate. Aspiration alone, without catheter placement, might lead to cystic reaccumulating prior to SRS, or in further weeks after SRS, before the BM has become nonviable, since the fluid is only being drained on the day of surgery. In this respect, placement of an intracystic reservoir before SRS might be considered as an useful strategy, as it allows for needle aspiration of cystic contents at multiple timepoints before and after SRS[ (Park et al., 2021)]. The rate of tumor size reduction after Ommaya was related to the placement of the tip inside the tumor, with a more favorable outcome when the tip was placed in the center (mean reduction 58%) as compared with deep (42.6%) and shallow (7.7%) [ (Oshima et al., 2017)]. Other factors, including the distance from the brain surface, type of primary cancer, homogeneity of the cyst, and existence of a tumor septum on imaging did not impact the rate of success [ (Oshima et al., 2017)].

Special caution needs to be taken when considering evolution of the tumor volume between cyst aspiration and SRS, as there is little literature on the topic[18]. It is important that the surgical wound experiences proper healing before SRS is performed. Additionally, one should bear in mind that for lesions close to the surface, lower isodose lines may cross with the scalp and interfere with incision healing. Complications of stereotactic cyst aspiration include hemorrhage, neurological deficits, seizure and infection [ (Lunsford and Martinez, 1984)]. Mortality rates in large series are less than 1% and complication rates vary between 0 and 7% [ (Lunsford and Martinez, 1984), (Bernstein and Parrent, 1994)]. Tumor volume reduction allows higher doses of radiation treatment and has a lower risk of radiation complications.

Based on the current literature, some recommendations can be summarized as follows: the tip of Ommaya should be placed in the center of the cyst [ (Oshima et al., 2017)]; case selection should consider eloquent tumor location, neurologically symptomatic, significant comorbidities (patients high-risk for general anesthesia)[ (Park et al., 2021)]; SRS should be performed within 2–3 weeks after surgery[18] (Table 3).

**Table 3:** Recommendations for future practice.

the tip of Ommaya should be placed in the center of the cyst
case selection should consider:
• eloquent tumor location
• neurologically symptomatic,
• significant comorbidities (patients high-risk for general anesthesia)
SRS should be performed within 2–3 weeks after surgery

An open question remains regarding the utility of such procedure for infratentorial lesions. Moreover, the optimal timing between different steps (Ommaya placement, cyst aspiration and SRS) as well as the associated neuroimaging assessment remains to be further established. Subjective surgical factors include how much volumetric reduction is needed to produce relief of symptomatic mass effect.

Our meta-analysis has several inherent limitations. The first is related to the retrospective nature of the included series. The second is the overall limited number of cases. A third limitation is that the follow-up in these patients is rather limited due to their primary disease and oncological outcome.

## 5 Conclusions

The combined treatment of Ommaya placement, cyst aspiration, and SRS in cystic BMs yields better local control as compared to stereotactic aspiration plus SRS alone. While the Ommaya approach did show a higher complication rate, these were generally minor and manageable. For the stereotactic aspiration group, complication rates were lower, but more serious (two deaths).

The overall rate of further intervention is similar between procedures. The Ommaya, cyst aspiration, and SRS treatment modality should be considered for patients with large cystic BMs who are not candidates for surgical resection or upfront SRS due to tumor size, location, or other comorbidities.

The use of an Ommaya reservoir allows for repeated aspiration, enhancing the effectiveness and flexibility in the timing of subsequent SRS. Careful patient selection and timely intervention are necessary to optimize patient outcomes.

## Ethical approval

No ethical approval was required for this meta-analysis of previously published data.

## Author contributions

All authors contributed to the study conception and design. Article review, article selection, and meta-analysis were performed by David Peters, Florentina Trandafirescu, and Constantin Tuleasca. The first draft of the manuscript was written by David Peters, Florentina Trandafirescu, and Constantin Tuleasca and all authors commented on previous versions of the manuscript. All authors read and approved the final manuscript.

No ethical approval was required for this meta-analysis of previously published data.

## Availability of data and materials

Not applicable.

## AI

No AI was used for this study.

## Funding

The authors declare that no funds, grants, or other support were received during the preparation of this manuscript.

## Declaration of competing interest

The authors declare that they have no known competing financial interests or personal relationships that could have appeared to influence the work reported in this paper.
